# Tracking the Transition from Pericyclic to Pseudopericyclic Reaction Mechanisms Using Multicenter Electron Delocalization Analysis: The [1,3] Sigmatropic Rearrangement

**DOI:** 10.1021/acs.jpca.1c06620

**Published:** 2021-09-11

**Authors:** Álvaro Pérez-Barcia, Ángeles Peña-Gallego, Marcos Mandado

**Affiliations:** Department of Physical Chemistry, University of Vigo, Lagoas-Marcosende s/n, 36310 Vigo, Spain

## Abstract

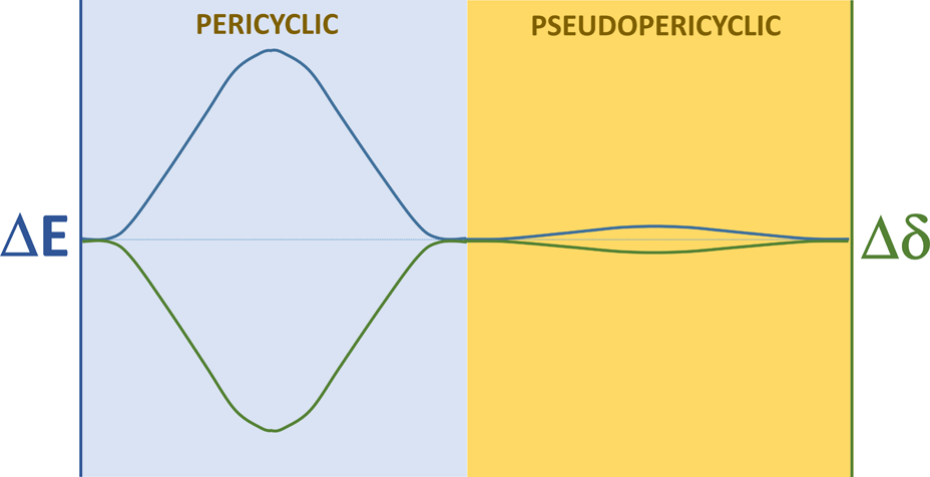

Herein, the power of multicenter electron delocalization analysis to elucidate the intricacies of concerted reaction mechanisms is brought to light by tracking the transition of [1,3] sigmatropic rearrangements from the high-barrier pericyclic mechanism in 1-butene to the barrierless pseudopericyclic mechanism in 1,2-diamino-1-nitrosooxyethane. This transition has been progressively achieved by substituting the migrating group, changing the donor and acceptor atoms, and functionalizing the alkene unit with weak and strong electron-donating and electron-withdrawing groups. Fourteen [1,3] sigmatropic reactions with electronic energy barriers ranging from 1 to 89 kcal/mol have been investigated. A very good correlation has been found between the barrier and the four-center electron delocalization at the transition state, the latter calculated for the atoms involved in the four-centered ring adduct formed along the reaction path. Surprisingly, the barrier has been found to be independent of the bond strength between the migrating group and the donor atom so that only the changes induced in the multicenter bonding control the kinetics of the reaction. Additional insights into the effect of atom substitution and group functionalization have also been extracted from the analysis of the multicenter electron delocalization profiles along the reaction path and qualitatively supported by the topological analysis of the electron density.

## 1 Introduction

Multicenter electron delocalization indices (MCIs),^1−3^ a quantum chemical tool based on the *n*-electron density function, were proposed 15 years ago as a powerful tool to characterize aromaticity in transition states (TSs) of pericyclic reactions.^4^ The great sensitivity of MCIs for detecting changes in the electron delocalization patterns within a reacting system was demonstrated. Thus, MCIs emerged as a very useful complementary or even alternative tool to molecular orbital (MO) correlation diagrams for characterizing reaction mechanisms, overcoming the problems inherent to interpretations based on MOs, which are not invariant to MO transformations or accessible when more accurate post-SCF methods are required.

Unfortunately, during the years following the publication of this guiding work,^4^ the applications of MCIs in characterizing concerted mechanisms in chemical reactions have been scarce.^5,6^ This is partly due to the existence of other valuable quantum chemical tools based on the quantum chemical topology, such as the electron localization function (ELF)^7−9^ or the topological analysis of electron density critical points.^10^ The former is routed on the two-electron probability density,^7^ whereas the latter is based on the one-electron density so that both are also invariant to MO transformations. These topological approaches based on electron densities have the advantage of providing a direct link with experiments as this property is experimentally observable and can be obtained from diffraction techniques. On the contrary, MOs are mathematical objects that allow the construction of determinantal wavefunctions, and they are nonunique and cannot be observed experimentally. As mentioned above, MO correlation diagrams can only be analyzed at single-determinant levels such as Hartree–Fock or Kohn–Sham density functional theory (DFT). On the other hand, topological approaches provide unique structures characterized by means of different kinds of critical points directly related to the bonding, allowing us to track bonding rearrangements along a reaction path. A special mention should be made of the bonding evolution theory (BET),^11^ where the ELF is combined with Thom’s catastrophe theory,^12^ providing a robust tool for monitoring the bonding rearrangement along a reaction path (see, for instance, ref (13) and references therein). As was shown very recently for the particular case of Diels–Alder reactions, a key step in the BET analysis is the identification of elementary catastrophes along the reaction path.^14^

The ELF is probably the most powerful tool to investigate chemical bonding in terms of Lewis bonding theory since it provides a real-space representation of the electron localization, allowing us to visualize bonds and lone electron pairs.^15^ However, tracking multicenter bonding or, more specifically, aromaticity requires, in principle, the analysis of the *n*-electron probability density, *n* being the number of atoms involved in the bonding. Aromaticity scales based on indices derived from the two-electron probability density have been proposed in the literature.^16,17^ However, they require the use of a reference system,^16^ the σ–π partition of the MO space,^16^ or the selection of specific atom pairs,^17^ preventing their application in many chemical reactions. In the case of the ELF, aromaticity may also be analyzed by means of bifurcation points between bond pairs arising from the π contribution to the ELF (ELF_π_).^18^ Unfortunately, for chemical reactions that evolve through a nonplanar structure, the σ–π partition of the electron density is not feasible, and the ELF might not be reliable in quantifying changes in the multicenter delocalization/aromaticity along the reaction path.

In this work, the application of MCIs to the study of concerted mechanisms is revived through the evaluation of one of the most controversial concepts in synthetic organic chemistry, the pericyclic or pseudopericyclic character of certain chemical reactions that take place in an apparently concerted fashion. Pericyclic reactions were defined by Woodward and Hoffmann as “reactions in which all first-order changes in bonding relationships take place in concert on a closed curve”.^19^ Woodward–Hoffmann rules, applying the MO theory, predict whether a pericyclic reaction is allowed or forbidden based on the number of electrons involved. However, a few years later, Lemal recognized a series of reactions that seemed to disobey the topological criteria established by the Woodward–Hoffmann rules. These reactions were named pseudopericyclic and defined as “concerted transformations in which the primary changes in bonding encompass a cyclic array of atoms, at one (or more) of which nonbonding and bonding atomic orbitals interchange roles”.^20,21^ An important feature of pseudopericyclic reactions is that they are always allowed by orbital symmetry, regardless of the number of electrons involved. In this sense, chemists have been taking advantage of the versatility of these reactions in the search for new synthetic routes.

Although the definition seems simple, the differentiation between pericyclic and pseudopericyclic reactions is not a trivial matter. The scope of pseudopericyclic reactions has been deeply analyzed, both experimentally and computationally.^22−37^ In addition to the fact that they always allow orbital symmetry, other characteristics that have been indicated for these reactions are low, or even nonexistent, barriers (very interesting in synthetic design), and planar, or close to planar, TSs. The aromatic character of the TS has been also employed in several studies as the difference between pericyclic and pseudopericyclic reactions.^38−45^ The orbital disconnection, characteristic of pseudopericyclic reactions, seems to prevent this aromaticity. However, an aromatic TS does not always imply the impossibility of a pseudopericyclic reaction.^46^ For this reason, the pericyclic or pseudopericyclic process must be studied as a whole and not only at the TSs.^47^

As aromatization affects magnetic properties, magnetic susceptibility, anisotropy, or nucleus-independent chemical shift it has traditionally been used to differentiate between pericyclic and pseudopericyclic reactions.^40,48^ However, aromaticity or, more concretely, a concerted bonding rearrangement is, in fact, a clear manifestation of multicenter electron delocalization, and therefore, MCIs seem to be a more appropriate tool for characterizing the pericyclic/pseudopericyclic character.^4^ Moreover, neither the definition of a reference system nor the partition into σ and π contributions is required with MCIs, which, in this particular case, entail important advantages over the ELF analysis mentioned above.

**Scheme 1:**
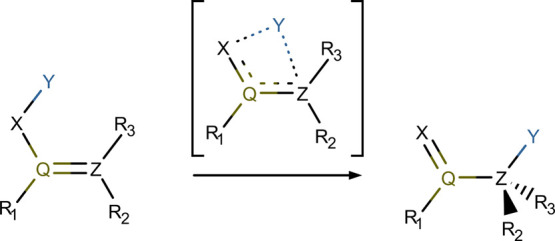
General Scheme of a [1,3] Sigmatropic Rearrangement

An example of a reaction where the pericyclic or pseudopericyclic character may be found depending on the kind of atoms involved is the [1,3] sigmatropic rearrangement (see Scheme 1 for a schematic representation, which includes also the atomic nomenclature employed in this work). Birney and co-workers studied different [1,3] sigmatropic rearrangements using MO theory.^26,27^ In particular, they characterized the nitroso group migration in the nitrosation of amides as pseudopericyclic in contrast to the well-known pericyclic rearrangement of the methyl group in 1-butene. In this work, we apply our multicenter electron delocalization analysis to a large set of [1,3] rearrangements ranging from the high-barrier pericyclic mechanism in 1-butene to the “ideal” barrierless pseudopericyclic mechanism in 1,2-diamino-1-nitrosooxyethane, including also the nitroso group migration studied in ref (26).

## 2 Methods

**Scheme 2:**
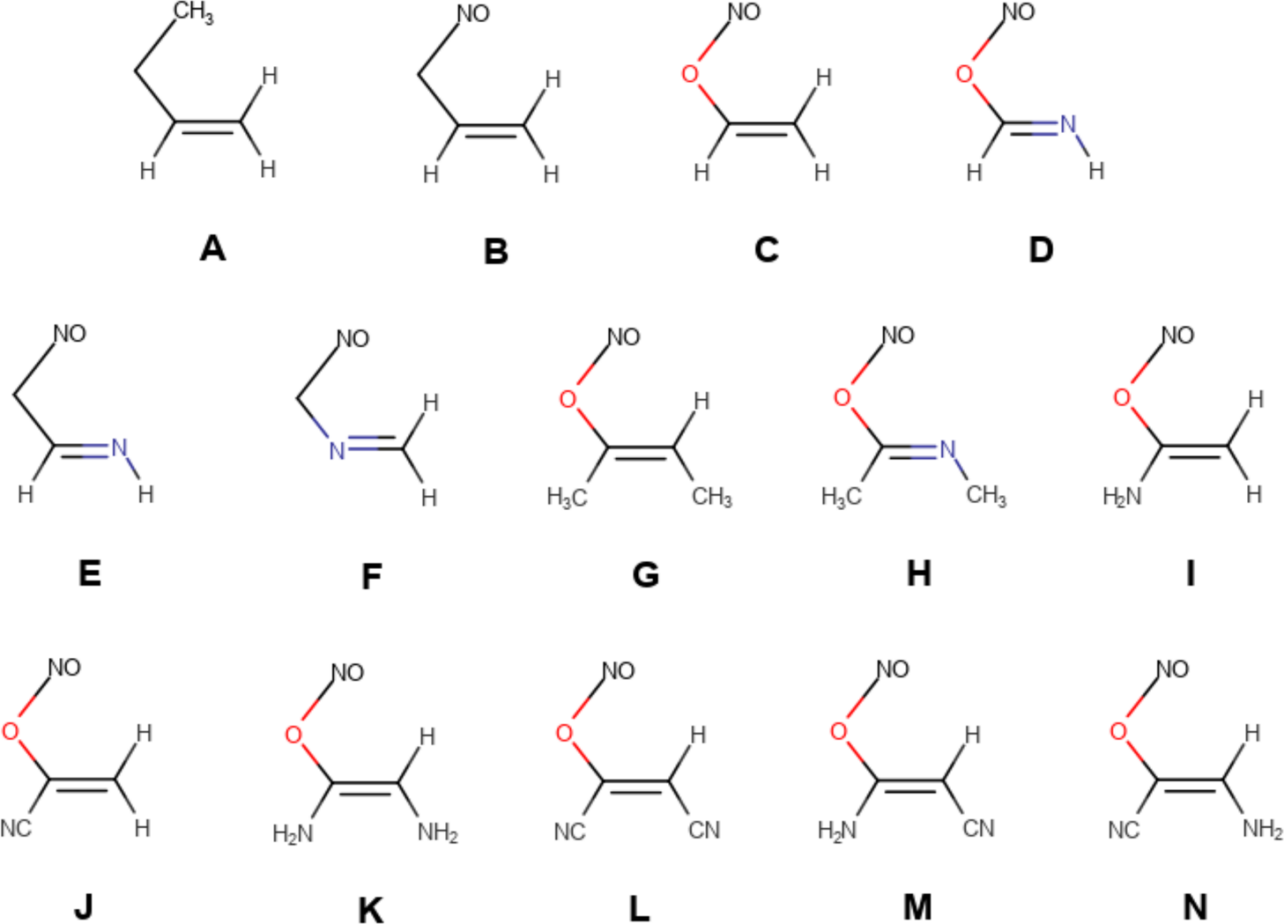
Nomenclature Employed for the [1,3] Sigmatropic Rearrangements Studied

The reactants considered in our study are shown in Scheme 2. The set comprises 14 systems which differ in the migrating group Y (methyl for **A** and nitroso for the remaining ones), the X atom (carbon for **A**, **B**, **E**, and **F** and oxygen for the remaining ones), and the Z atom (nitrogen for **D**, **E**, and **H** and carbon for the remaining ones). In system **F**, the imine group has been inverted with respect to system **E** so that nitrogen is not the Z atom but the Q atom. Finally, the effect of functionalizing the alkene/imine unit on the pericyclic/pseudopericyclic character is investigated in systems **G–N** by introducing a weak activating group, methyl, a strong activating group, amino, and a strong deactivating group, cyano, at the Q and Z positions. The structures of reactants, TSs, and products are shown in the Supporting Information.

All the geometry optimizations and electron density calculations have been carried out within the framework of DFT at the B3LYP/6-31G(d,p) level with the Gaussian 09 software package.^49^ AIMALL 17.11.14 program^50^ has been employed for the topological analysis of the electron density, presented at the end of the next section. Herein, the analysis has been limited to the characterization of the ring critical point (RCP) formed along the reaction path as well as the bond critical points (BCPs) destroyed and formed at the reactant and product structures, respectively. Two-center delocalization indices, δ_2_, and four-center delocalization indices, δ_4_, have been calculated with the NDELOC program.^3^ The former gives the measure of the bond order for a given atom pair, whereas the latter measures the aromaticity in a four-center ring. For a theoretical background on electron delocalization indices and their application to the study of concerted reactions, the reader is referred to refs (1)–^4^.

All the TSs found for the [1,3] sigmatropic rearrangements have been connected to the reactant and product structures by following the corresponding intrinsic reaction coordinate (IRC). A large number of intermediate structures along the reaction path have been selected in each case, and the corresponding energies and electron densities have been determined. Both the topological and multicenter bonding analyses have been performed for each intermediate structure in order to track the multicenter bonding along the reaction paths. Energy barriers are given here as the difference between the TS and reactant electronic energies. No zero-point vibrational energy correction has been added since MCIs can correlate only with pure electronic effects. Thus, the energy barriers calculated in this work are not directly comparable with those reported in previous studies^26,27^ for the corresponding reactions.

## 3 Results and Discussion

First, we will perform a general analysis of the relation between the energy barrier and the electron delocalization along both the X–Y–Z–Q chain and the X–Y bond order. Subsequently, the effect of the migrating group, the nature of the atoms forming the ring adduct, and the functionalization of the alkene/imine unit will be discussed separately.

**Figure 1:**
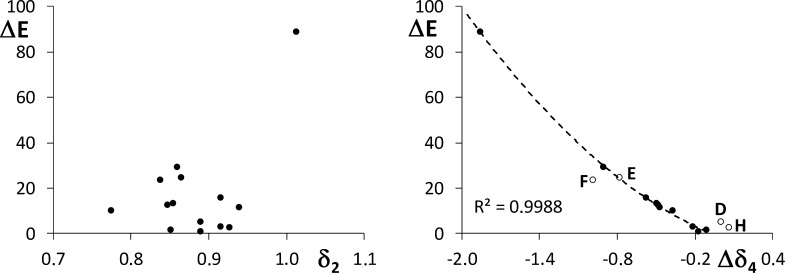
Left: reaction energy barrier vs the two-center delocalization index of the X–Y bond at the reactant structure. Right: reaction energy barrier vs the change in the four-center delocalization index of the X–Y–Z–Q chain, the TS minus the reactant. Second-order polynomial regression line (Δ*E* vs Δδ_4_) is shown (dotted line) together with the corresponding regression coefficient. Energies in kcal/mol and delocalization indices in au.

The electronic energy barriers are collected in Table 1. As can be observed, the values obtained for the barriers range from the high value of **A**, 88.7 kcal/mol, to the negligible value of **K**, 1.0 kcal/mol. Remarkably, the barrier for the migration of a methyl group (system **A**) is much larger than the barriers obtained for the migration of a nitroso group (systems **B–N**). Looking at the values of δ_2_ and δ_4_ for **A** and **B**, one could think the large barrier lowering is due to a weakening in the bond formed between the migrating group and the methyl carbon (the X–Y bond), a decrease in the four-center electron delocalization in the four-centered ring adduct formed at the TS, or even a combination of both effects. However, the X–Y bond orders obtained for the remaining systems clearly indicate that the barrier is not governed by the X–Y bond strength. Thus, no correlation has been found between δ_2_ and Δ*E*, as can be observed in Figure 1. On the contrary, the very good correlation found between δ_4_ and Δ*E*, also shown in Figure 1, indicates that the barrier strongly depends on the delocalization within the ring adduct at the TS, more precisely, on the change of delocalization with respect to the reactant, Δδ_4_. As highlighted in the right plot of Figure 1, the structures containing an imine group instead of an alkene deviate slightly from the rest, with the exception of **E**. More interesting is the fact that they show an opposite deviation depending on the position of the imine nitrogen (Q or Z), which reflects the important role played by the double bond in the [1,3] rearrangement.

**Table 1:** Reaction Energy Barrier, Δ*E*, Four-Center Delocalization Index, δ_4_, for the X–Y–Z–Q Chain at the TS, and Two-Center Delocalization Index, δ_2_, for the X–Y Bond at the Reactant^a^

	Δ*E*	δ_4_	δ_2_
**A**	88.7	–1.874	1.013
**B**	29.1	–1.071	0.860
**C**	15.7	–0.770	0.915
**D**	5.2	–0.203	0.890
**E**	26.5	–0.898	0.864
**F**	23.7	–1.033	0.837
**G**	11.6	–0.676	0.939
**H**	2.7	–0.135	0.927
**I**	3.3	–0.450	0.916
**J**	12.7	–0.691	0.847
**K**	1.0	–0.363	0.890
**L**	10.2	–0.538	0.774
**M**	2.0	–0.328	0.851
**N**	13.3	–0.663	0.855

a Energies in kcal/mol and delocalization indices in au.

Summarizing this general analysis, the data as a whole reflect the transition from a pericyclic rearrangement with a high energy barrier and high multicenter electron delocalization at the TS to a pseudopericyclic one with a negligible barrier and multicenter delocalization. Taking into account that these are the main criteria to discern between pericyclic and pseudopericyclic reactions, we can state that the transition occurs between these two reaction mechanisms.

### 3.1 Effect of Heteroatom Substitution

**Figure 2:**
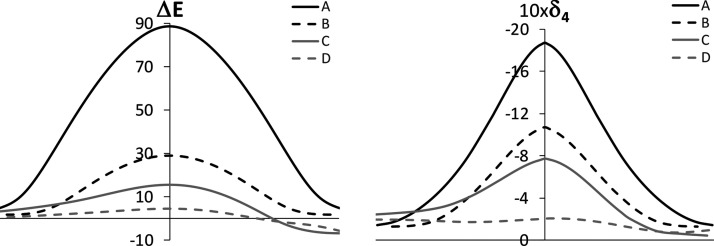
Comparison of the energy profile (left) and the four-center delocalization index profile (right) along the reaction coordinate of the [1,3] sigmatropic rearrangements for structures **A**, **B**, **C**, and **D**. Energies in kcal/mol and delocalization indices in au.

Figure 2 shows the comparison of the energy and four-center delocalization profiles along the IRC of the [1,3] rearrangement for **A**, **B**, **C**, and **D**. Herein, one can observe the effect of changing the nature of the atoms at different positions of the X–Y–Z–Q chain. Taking **A** as the reference system, the carbon atom at Y is replaced by nitrogen in **B**. As mentioned above, the four-center delocalization along the chain decreases significantly, which comes with a drastic drop in the energy barrier. An additional barrier lowering is induced in **C** by the substitution of the carbon atom at X by oxygen. In this case, the barrier lowering is 13.4 kcal/mol, which is proportional to the drop in the four-center delocalization, 0.3 au, compared with the change in **B** with respect to **A**, 0.8 au. The last change introduced in the chain is done in **D** with the substitution of the carbon atom at Q by nitrogen. This comes again with a barrier reduction of 10.5 kcal/mol and a decrease in the delocalization of 0.55 au. As mentioned above, systems containing an imine group instead of an alkene display small deviations in the correlation between multicenter delocalization and the energy barrier, and thus, there is a lack of proportionality here with respect to the changes observed for the pairs **A–B** and **B–C**. However, the important conclusion extracted from Figure 2 is that the very low barrier observed in **D** is associated with a flat profile of the four-center delocalization index along the IRC.

### 3.2 Effect of Methylation

The straight relation observed between the multicenter electron delocalization and energy barrier suggests that the control of this barrier may be achieved by tuning the electron delocalization within the X–Y–Z–Q chain. We have seen in the previous subsection how this can be done by introducing heteroatoms, which disrupt the synchronicity of the bonding rearrangement, reflected in the drop in the four-center electron delocalization. Another way to change this electron delocalization is through the functionalization of the alkene/imine unit at the Q and Z positions.

**Figure 3:**
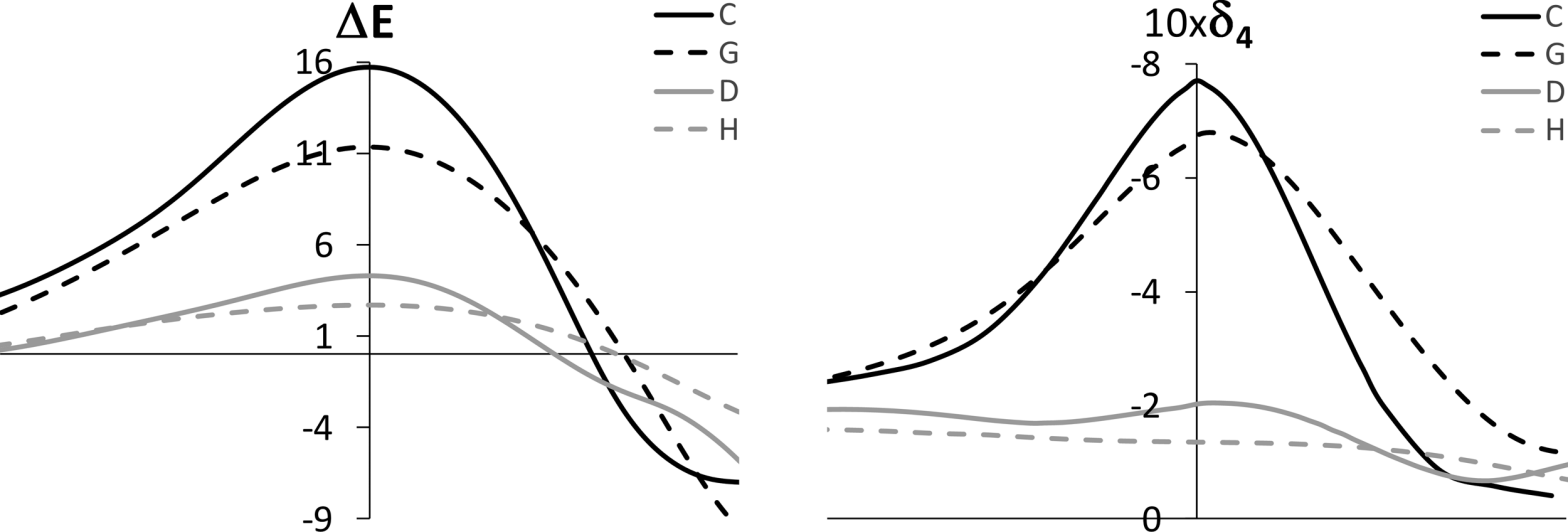
Comparison of the energy profile (left) and the four-center delocalization index profile (right) along the reaction coordinate of the [1,3] sigmatropic rearrangements for structures **C**, **G**, **D**, and **H**. Energies in kcal/mol and delocalization indices in au.

We start by introducing a weak electron-donating group. Methyl is expected to introduce little changes in the electron delocalization compared to strong electron-donating/-withdrawing mesomeric groups. In our study, **G** and **H** molecules correspond to the fully methylated **C** and **D** molecules. A comparison of the energy and multicenter delocalization profiles along the [1,3] rearrangements in these systems provides a perfect picture of the methylation effects. These profiles are shown in Figure 3. As can be observed, the methylation of **C** and **D** comes with reductions of 0.094 and 0.068 au in the four-center delocalization at the TS, respectively. These reductions are proportional to the changes in the energy barriers, which are 4.1 and 2.5 kcal/mol, respectively. Thus, even methyl, a weak electron-donating group, may introduce significant changes in the ring electron delocalization and, thus, in the energy barrier.

### 3.3 Effect of Strong Activating/Deactivating Groups

Looking for an ideal barrierless pseudopericyclic [1,3] rearrangement, we have functionalized the alkene unit in **C** with strong electron-donating/-withdrawing groups and analyzed the four-center electron delocalization in the X–Y–Z–Q chain. Thus, functionalizations with amino and cyano groups give rise to a series of molecules **I**–**N**. We have considered simultaneous functionalizations at the Z and Q positions (molecules **K**, **L**, **M**, and **N**) and the functionalization at the Q position only (molecules **I** and **J**), which, as will be shown below, is the most successful way to shift the [1,3] rearrangement of **C** to a more pseudopericyclic character.

**Figure 4:**
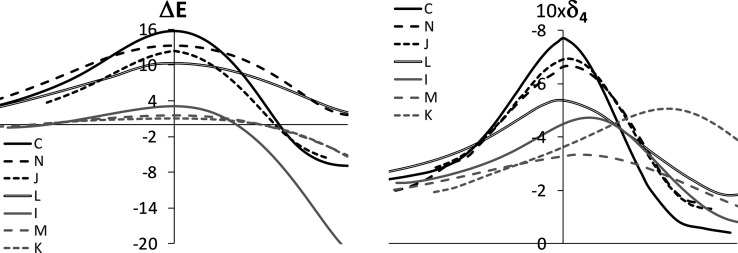
Comparison of the energy profile (left) and the four-center delocalization index profile (right) along the reaction coordinate of the [1,3] sigmatropic rearrangements for structures **C**, **N**, **J**, **L**, **I**, **M**, and **K**. Energies in kcal/mol and delocalization indices in au.

In Figure 4, one can see a comparison of the energy and four-center delocalization profiles along the IRC for systems **C** and **I–N**. Both the delocalization and energy profiles show the same relative order in the TS region, with the exception of the pairs (**J**, **N**) and (**K**, **M**), where the differences are very small and the trend seems to be reversed (see also the values in Table 1). However, the differences in these pairs are negligible, both in energy and electron delocalization profiles, and the trend is recovered in the pair (**J**, **N**) when Δδ_4_ is considered instead of δ_4_.

What could seem an unexpected result a priori is the general decrease of the ring electron delocalization and, thus, the lowering of the energy barrier for all the functionalized molecules. This result is independent of the electron-donating or electron-withdrawing nature of the substituent and the position where the functionalization takes place. However, the effect is significantly different in magnitude, with the amino-functionalization at the Q position being the one that shifts the mechanism the most to an ideal pseudopericyclic one. Thus, the energy barriers for **I**, **M**, and **K** are 3.3, 2.0, and 1.0 kcal/mol, respectively, which may be considered negligible values at room temperature. In the three cases, the change in δ_4_ from the reactant to the TS is also negligible. However, the change with respect to the product is significantly larger (see the full profiles shown in the Supporting Information), which is also in line with the much higher energy barriers calculated for the reversed reactions, 33.2, 28.9, and 15.8 kcal/mol for **I**, **K**, and **M**, respectively.

There are some other features that point out a change in the rearrangement mechanism for the amino-functionalized molecules at the Q position. A more or less pronounced maximum of δ_4_ appears after the TS, which is clearly displaced to the product structure, contrary to the remaining systems where a well-pronounced maximum is located at the TS. The most remarkable example is **K**. In this case, the value at the maximum is close in magnitude to the value at the maximum displayed by **L** (see Figure 4), whereas the value at the TS is much lower. This means that a ring adduct is also formed during the [1,3] rearrangement of these amino-functionalized molecules, but it does not rule the energy barrier as it is formed well after the TS.

**Figure 5:**
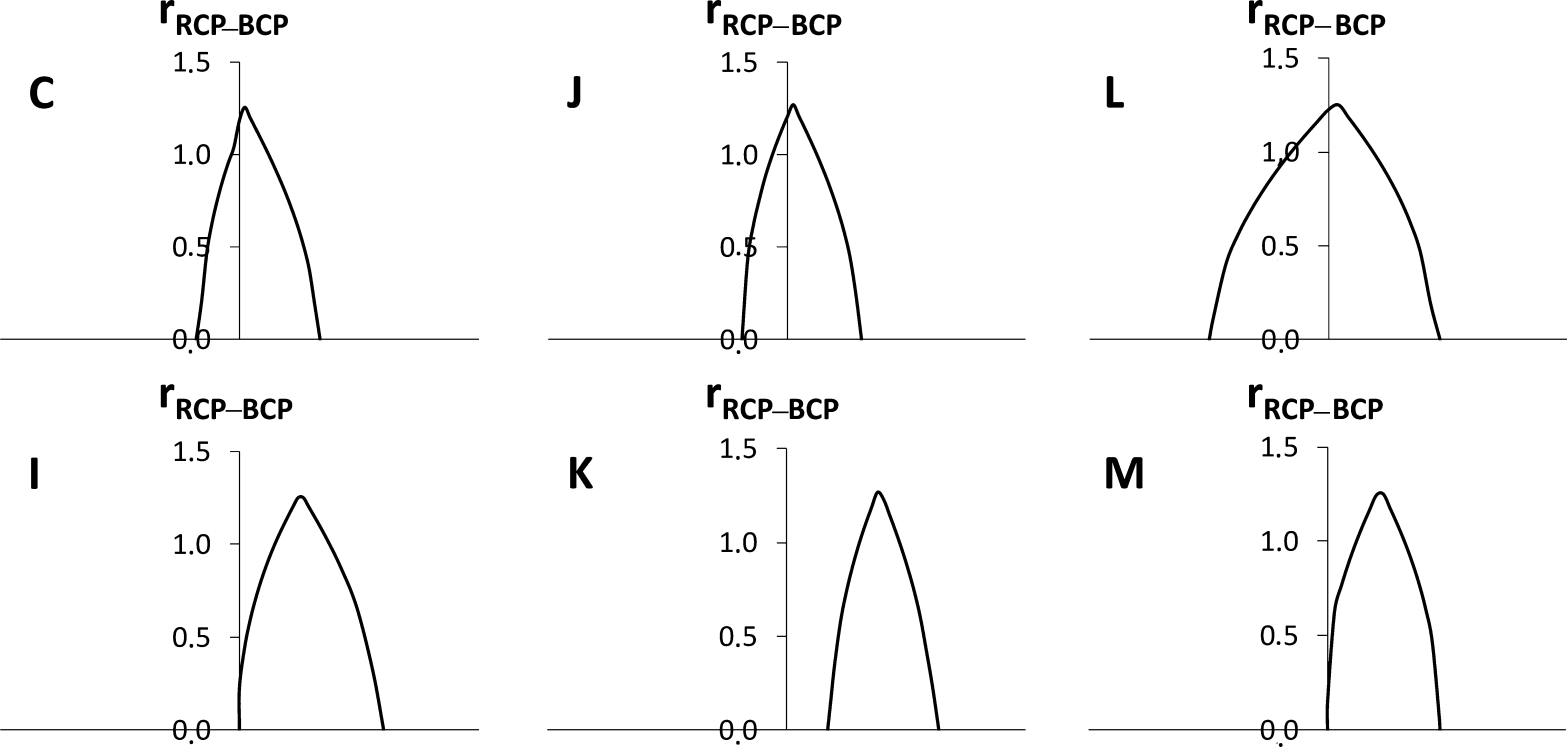
Evolution of the RCP associated with the ring formed by the X, Y, Z, and Q atoms along the reaction coordinate of the [1,3] sigmatropic rearrangements for structures **C**, **J**, **L**, **I**, **K**, and **M**. The distances (in au) between the RCP and the Y–Z BCP (on the right of the maximum) and the RCP and the X–Y BCP (on the left of the maximum) are represented so that the creation of the RCP at the Y–Z BCP, its migration from the Y–Z BCP to the X–Y BCP, and its annihilation at the X–Y BCP may be observed.

In order to check that the shift in the ring formation to the product structure is not an artifact of our multicenter delocalization analysis, a topological analysis of the electron density has also been performed. The analysis has been focused exclusively on the characterization of the RCP associated with the X–Y–Z–Q chain. Since the nature of the atoms involved is not the same for all the molecules studied, RCP properties such as the electron density, the Laplacian of the electron density, or the energy density cannot be compared directly. Thus, we have just followed the evolution of the RCP along the IRC, characterizing the points where the RCP emerges and collapses with the Z–Q and X–Y BCPs, respectively. To visualize in a simple fashion these singular points and the intermediate region where the RCP exists, the minimum distance from the RCP to these BCPs, r_RCP–BCP_, is represented along the reaction path. One can see these representations in Figure 5 for the [1,3] rearrangements of six representative molecules. **C**, **J**, and **L** are examples where the maximum of δ_4_ is located at the TS, and **I**, **M**, and **K** are those where this maximum is shifted to the product. Points with *r*_RCP–BCP_ > 0 delimitate the region where the RCP exits. As can be observed, the plots are centered at the TS and extend equally to the reactant and product structures in **C**, **J**, and **L**, whereas the plots are confined within the right side of the reaction in **I**, **M**, and **K**. This means that the RCP is formed right after the TS is overcome and its migration progresses as the energy drops to the product.

In Figure 4, the energy and multicenter delocalization profiles for cyano-functionalized molecules at the Q position (**J**, **L**, and **N**) are also shown. Herein, cyano-functionalization at Z and Q exerts a similar effect on the barrier and delocalization, reducing the former by about 2–3 kcal/mol in each case (compare the barriers for **J** and **L** in Table 1). Additional amino-functionalization at Z (molecule **N**) exerts a little effect on the barrier and delocalization, with a slight increase in the former (0.6 kcal/mol). This little effect is, however, opposite to that observed for the amino-functionalized molecules **I** and **M**, where the additional cyano-functionalization at Z (molecule **M**) slightly reduces the barrier (1.3 kcal/mol).

## 4 Conclusions and Future Prospects

In this work, a quantitative tool to characterize concerted reaction mechanisms as pericyclic or pseudopericyclic has been tested. This tool is based on the direct measurement of multicenter electron delocalization through the calculation of MCIs. These indices were introduced some years ago as a valuable aromaticity indicator for TSs of pericyclic reactions, but their application to the study of concerted reaction mechanisms has certainly been scarce. However, this work tries to revive the use of MCIs for studying concerted processes. Thus, the power of this simple quantum chemical tool to monitor the multicenter electron delocalization, not only at the TSs but also along the reaction paths, has been revealed. A series of 14 [1,3] sigmatropic reactions, ranging from high-barrier to barrierless processes, have been investigated herein. A very good correlation between MCIs and energy barriers has been found, relating unambiguously the experimentally observed connection between the high/low barriers and pericyclic/pseudopericyclic character, with the cyclic electron delocalization being an experimentally inaccessible quantity. Additionally, the MCI analysis allows us to evaluate quantitatively the effect of atom substitution or group functionalization on the cyclic electron delocalization within the ring adduct formed during the reaction and, thus, on the displacement of a given concerted process to a more or less pseudopericyclic character. Its invariance under MO transformations, its nonlocal nature, and the need to not introduce reference systems or perform σ/π partitions make the MCI analysis superior to others, such as the ELF or the analysis of electron density critical points, in the characterization of concerted processes. MCIs can complement studies where the MO connection/disconnection was employed to elucidate pericyclic/pseudopericyclic mechanisms; for instance, the [3,3] sigmatropic rearrangements studied in ref (37), where a pericyclic rearrangement proceeds via the chair transition structure and a pseudopericyclic one through the boat transition structure. MCIs can provide very useful information about the role played by the cyclic electron delocalization in the change of the mechanism. Therefore, future effort must be oriented to the integration of MCIs as a regular tool for the study of controversial cases since they may provide a pericyclic/pseudopericyclic scale.

## Supporting Information Available

Energy and four-center delocalization index profiles along the reaction coordinate for the [1,3] sigmatropic rearrangements in the 14 reactions studied; reactant, TS, and product structures for the [1,3] sigmatropic rearrangements in the 14 reactions studied; and Cartesian coordinates for the reactant, TS, and product structures of the [1,3] sigmatropic rearrangements in the 14 reactions studied (PDF)
