# Supplementary material for: Tracking the Transition from Pericyclic to Pseudopericyclic Reaction Mechanisms Using Multicenter Electron Delocalization Analysis: The [1,3] Sigmatropic Rearrangement
Source: J Phys Chem A. 2021 Sep 11;125(37):8337–44. doi: 10.1021/acs.jpca.1c06620 (PMC8693182; doi:10.1021/acs.jpca.1c06620)
Supplement: Supplementary file 1 — jp1c06620_si_001.pdf [file jp1c06620_si_001.pdf]

Tracking the Transition from Pericyclic to Pseudopericyclic Reaction Mechanisms Using Multicenter Electron Delocalization Analysis: The [1,3] Sigmatropic Rearrangement

Álvaro Pérez-Barcia, Ángeles Peña-Gallego and Marcos Mandado*

Department of Physical Chemistry, University of Vigo, Lagoas-Marcosende s/n, 36310.Vigo, Spain

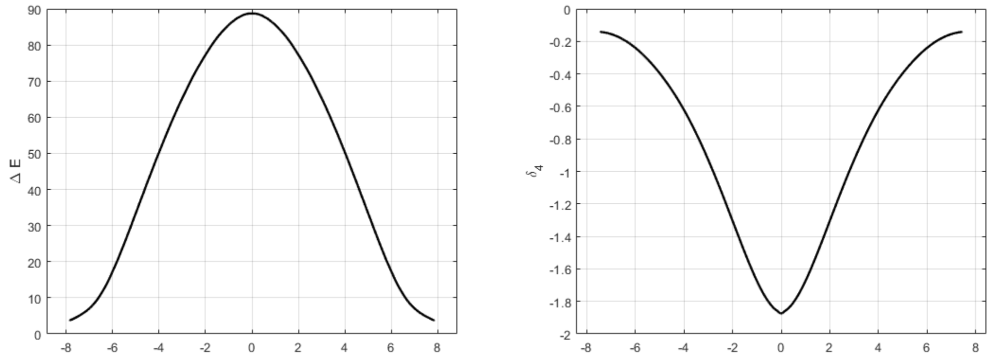

Figure S1. Energy profile (left) and the 4-center delocalization index profile (right) along the reaction coordinate of the [1,3] sigmatropic rearrangements for structures **A**. Energy in kcal/mol and delocalization index in au.

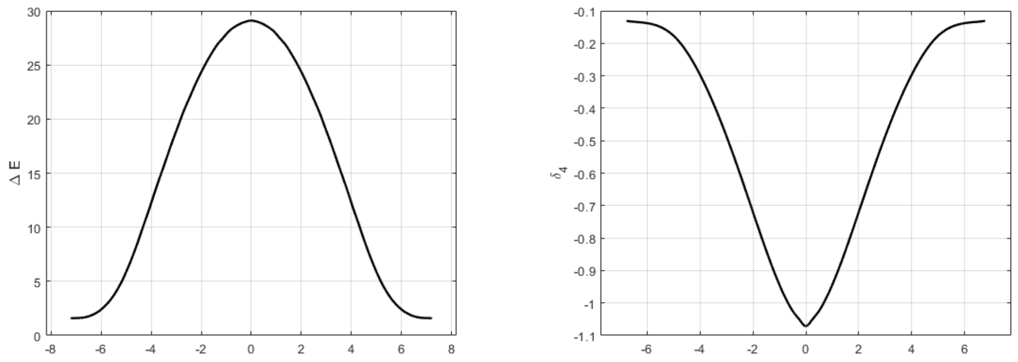

Figure S2. Energy profile (left) and the 4-center delocalization index profile (right) along the reaction coordinate of the [1,3] sigmatropic rearrangements for structures **B**. Energy in kcal/mol and delocalization index in au.

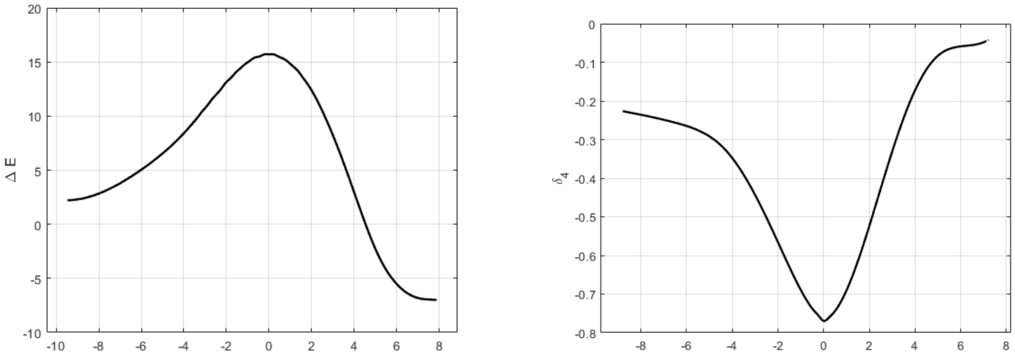

Figure S3. Energy profile (left) and the 4-center delocalization index profile (right) along the reaction coordinate of the [1,3] sigmatropic rearrangements for structures **C**. Energy in kcal/mol and delocalization index in au.

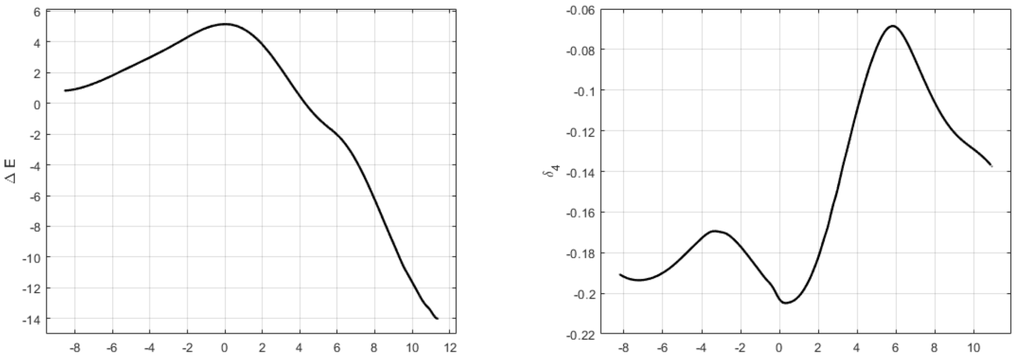

Figure S4. Energy profile (left) and the 4-center delocalization index profile (right) along the reaction coordinate of the [1,3] sigmatropic rearrangements for structures **D**. Energy in kcal/mol and delocalization index in au.

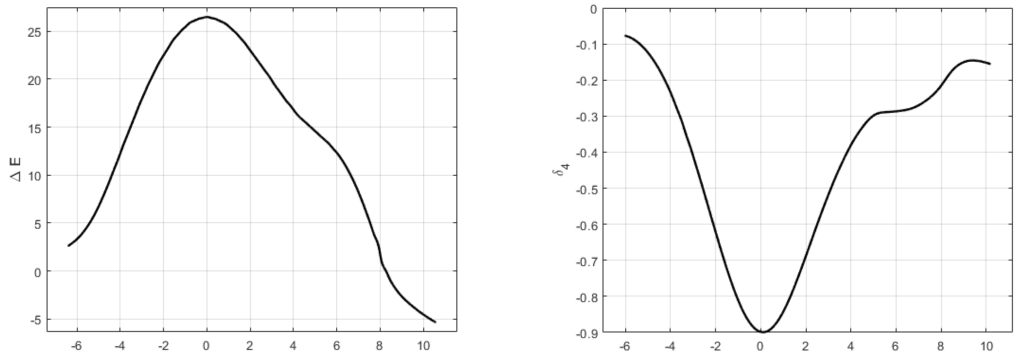

Figure S5. Energy profile (left) and the 4-center delocalization index profile (right) along the reaction coordinate of the [1,3] sigmatropic rearrangements for structures **E**. Energy in kcal/mol and delocalization index in au.

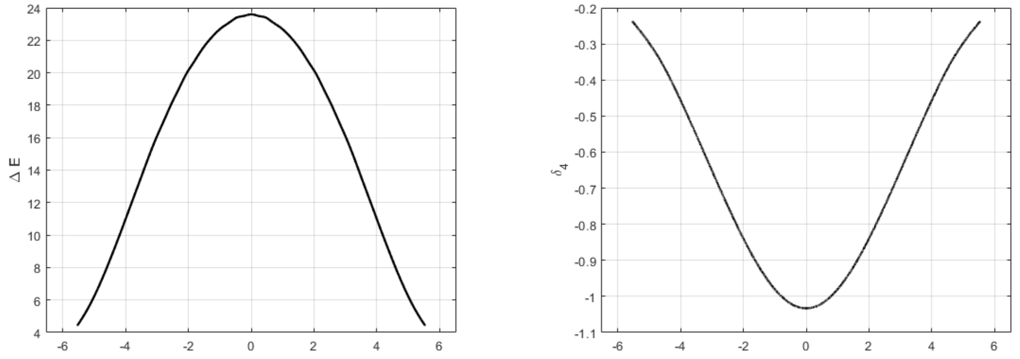

Figure S6. Energy profile (left) and the 4-center delocalization index profile (right) along the reaction coordinate of the [1,3] sigmatropic rearrangements for structures **F**. Energy in kcal/mol and delocalization index in au.

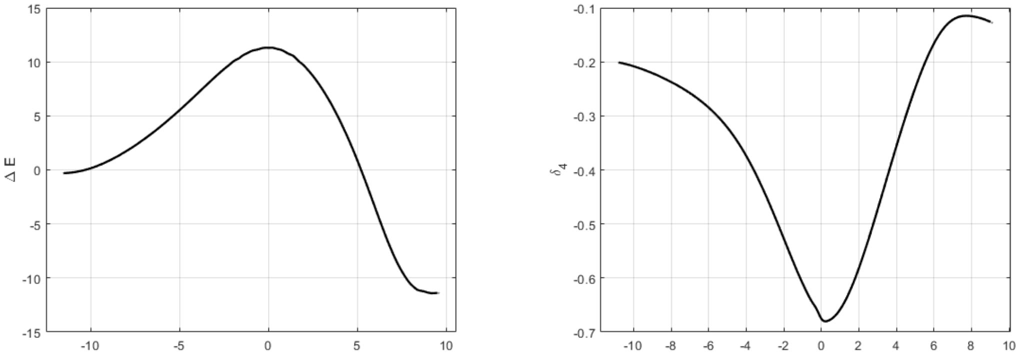

Figure S7. Energy profile (left) and the 4-center delocalization index profile (right) along the reaction coordinate of the [1,3] sigmatropic rearrangements for structures **G**. Energy in kcal/mol and delocalization index in au.

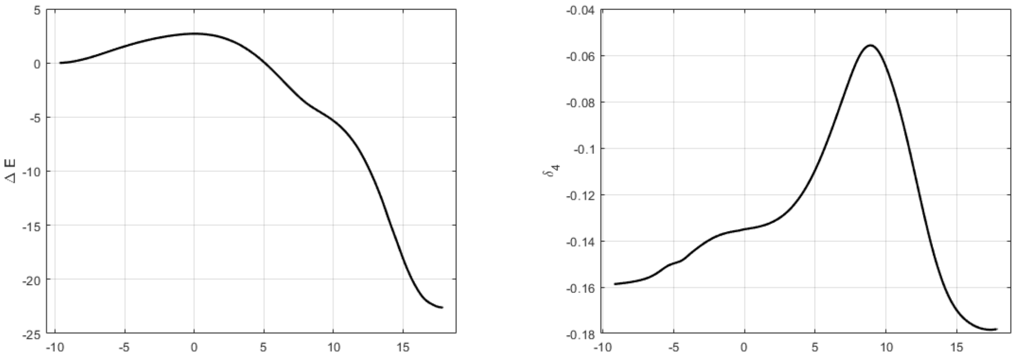

Figure S8. Energy profile (left) and the 4-center delocalization index profile (right) along the reaction coordinate of the [1,3] sigmatropic rearrangements for structures **H**. Energy in kcal/mol and delocalization index in au.

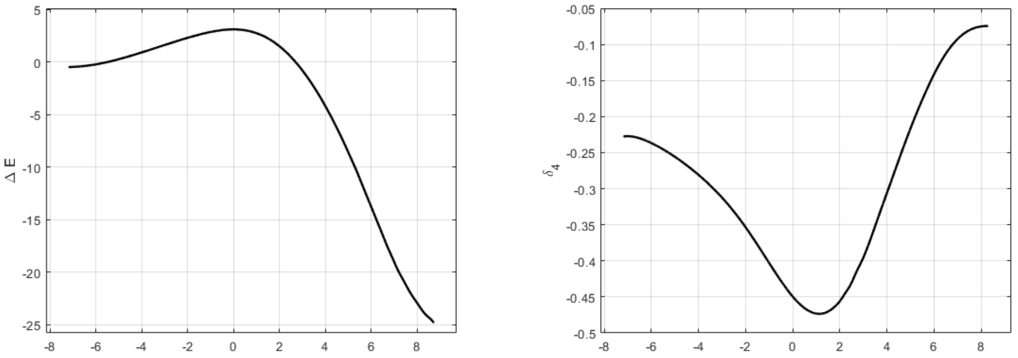

Figure S9. Energy profile (left) and the 4-center delocalization index profile (right) along the reaction coordinate of the [1,3] sigmatropic rearrangements for structures **I**. Energy in kcal/mol and delocalization index in au.

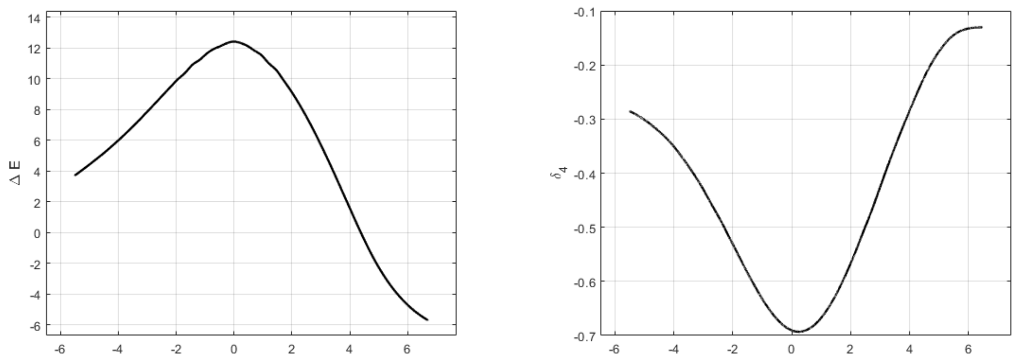

Figure S10. Energy profile (left) and the 4-center delocalization index profile (right) along the reaction coordinate of the [1,3] sigmatropic rearrangements for structures **J**. Energy in kcal/mol and delocalization index in au.

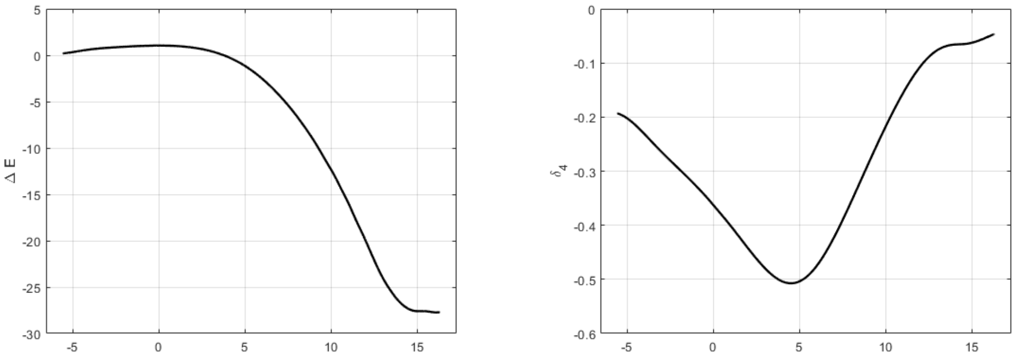

Figure S11. Energy profile (left) and the 4-center delocalization index profile (right) along the reaction coordinate of the [1,3] sigmatropic rearrangements for structures **K**. Energy in kcal/mol and delocalization index in au.

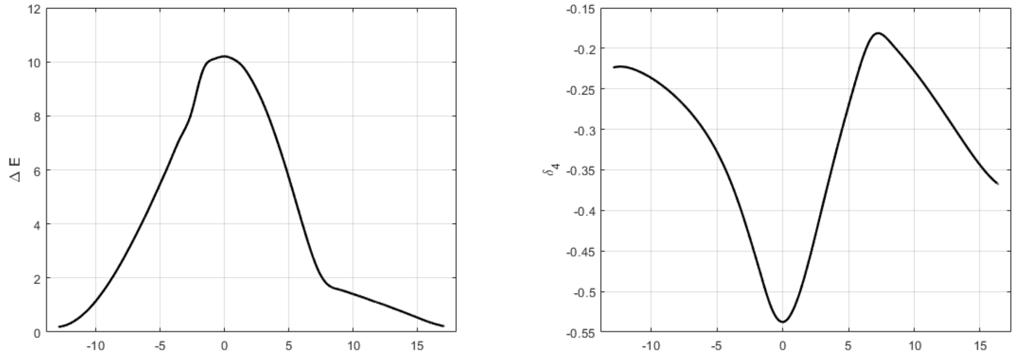

Figure S12. Energy profile (left) and the 4-center delocalization index profile (right) along the reaction coordinate of the [1,3] sigmatropic rearrangements for structures **L**. Energy in kcal/mol and delocalization index in au.

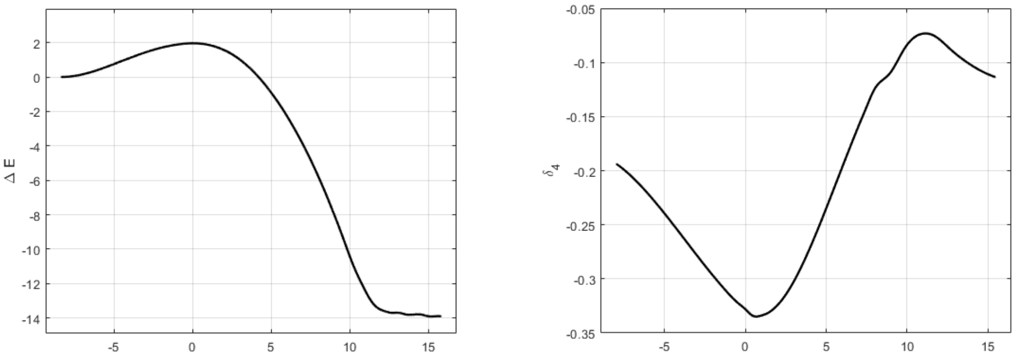

Figure S13. Energy profile (left) and the 4-center delocalization index profile (right) along the reaction coordinate of the [1,3] sigmatropic rearrangements for structures **M**. Energy in kcal/mol and delocalization index in au.

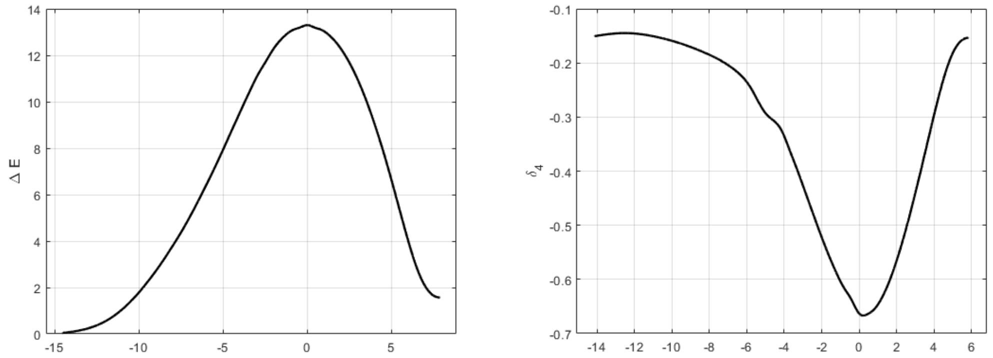

Figure S14. Energy profile (left) and the 4-center delocalization index profile (right) along the reaction coordinate of the [1,3] sigmatropic rearrangements for structures **N**. Energy in kcal/mol and delocalization index in au

A

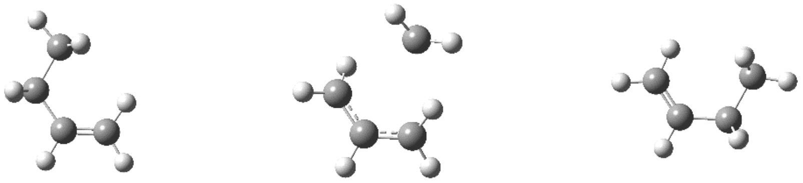

B

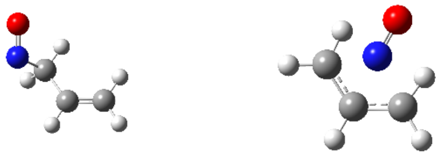

C

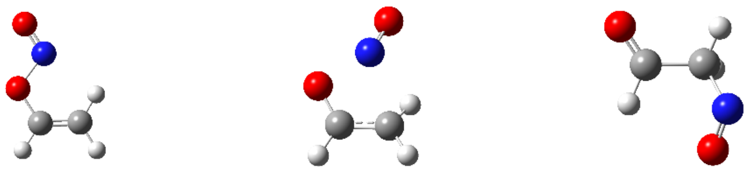

D

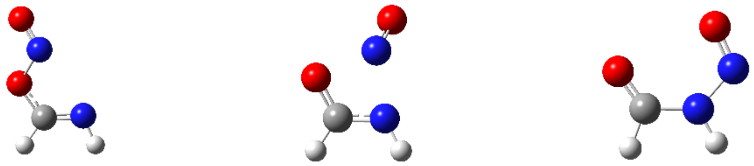

E

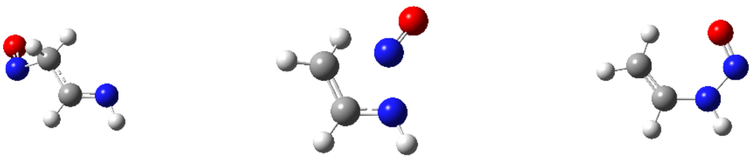

F

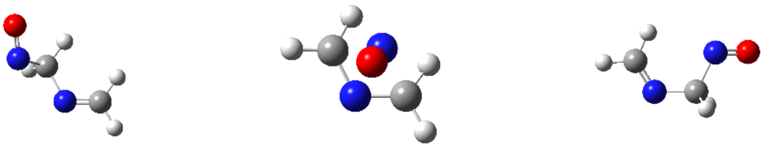

G

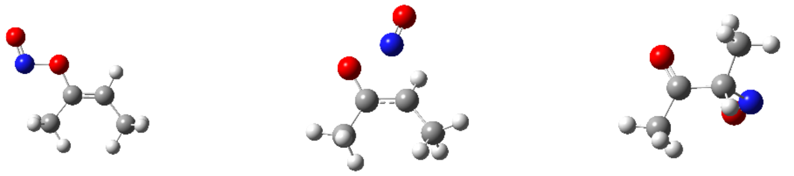

H

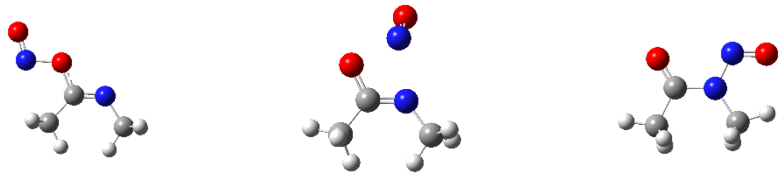

I

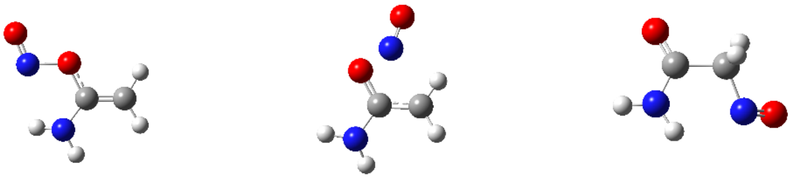

J

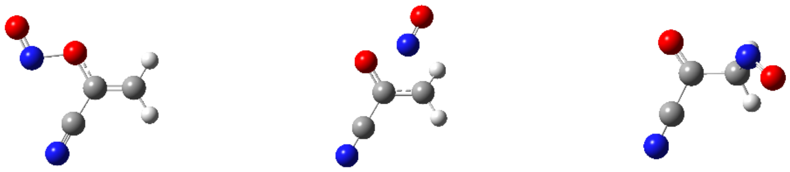

K

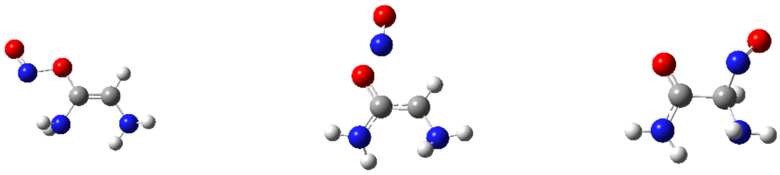

L

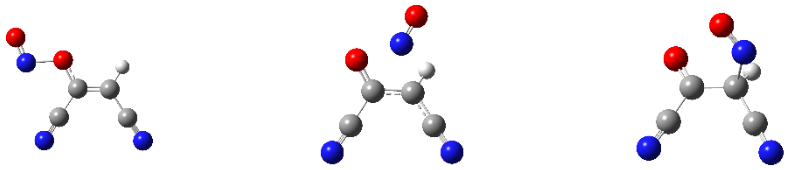

M

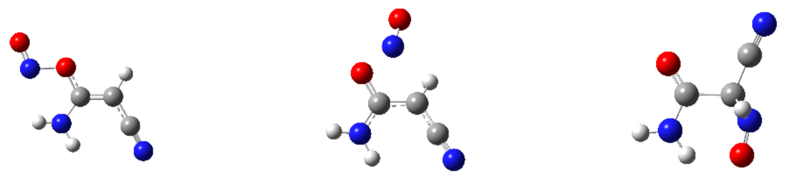

N

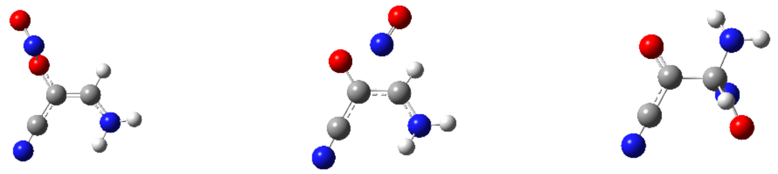

Figure S15. Representations from left to right of the reactant, transition state (TS) and product for each of the studied [1,3] sigmatropic rearrangements

Table S1. Cartesian coordinates of the reactant, TS and product for reaction A.

	Reactant			TS			Product		
	X	Y	Z	X	Y	Z	X	Y	Z
C	0.642888	0.721541	-0.000003	-0.591163	-1.231173	0.018053	0.855788	0.560348	0.000001
H	0.924562	1.331720	0.871055	-1.073995	-2.115955	-0.393305	-0.642888	0.721540	-0.000001
C	-0.855789	0.560349	0.000002	-1.202800	0.006568	-0.008842	-0.924560	1.331717	0.871059
H	-1.416582	1.495941	0.000007	-2.299272	0.018520	-0.039857	-1.453698	-0.575139	0.000000
C	-1.534962	-0.586982	-0.000001	-0.559151	1.232187	0.009163	-1.234127	-1.182097	-0.884391
H	-2.620669	-0.599083	-0.000001	-1.048308	2.121196	0.403927	-1.234123	-1.182097	0.884390
H	-1.041621	-1.554418	-0.000004	0.331725	1.383332	-0.574599	1.534965	-0.586982	0.000000
C	1.453696	-0.575139	0.000002	1.913413	-0.011937	-0.004741	2.620671	-0.599078	-0.000001
H	2.526509	-0.360288	0.000001	2.261605	-0.947375	0.419136	1.041626	-1.554419	0.000000
H	1.234122	-1.182094	0.884394	2.005557	0.068066	-1.082636	1.416580	1.495940	0.000002
H	1.234123	-1.182100	-0.884388	2.170522	0.871183	0.566181	-2.526510	-0.360286	0.000003
H	0.924557	1.331712	-0.871067	0.290370	-1.372836	0.619360	-0.924558	1.331715	-0.871063

Table S2. Cartesian coordinates of the reactant, TS and product for reaction B.

	Reactant			TS			Product		
	X	Y	Z	X	Y	Z	X	Y	Z
C	2.152124	-0.483176	-0.082717	0.790962	-1.221315	0.223024	0.790962	-1.221315	0.223024
H	2.077745	-1.332119	0.591963	0.226087	-1.326978	1.143578	0.226087	-1.326978	1.143578
C	1.176910	0.419055	-0.185308	1.335944	0.000000	-0.157933	1.335944	0.000000	-0.157933
N	-1.274828	0.336373	-0.357044	-0.992739	0.000000	-0.549283	-0.992739	0.000000	-0.549283
O	-2.055346	-0.555385	-0.119956	-1.903189	0.000000	0.169584	-1.903189	0.000000	0.169584
H	3.065152	-0.398928	-0.663806	1.112197	-2.143320	-0.249300	1.112197	-2.143320	-0.249300
H	1.274737	1.255334	-0.874989	1.990909	0.000001	-1.028930	1.990909	0.000001	-1.028930
C	-0.102532	0.359889	0.596335	0.790963	1.221315	0.223024	0.790963	1.221315	0.223024
H	-0.162214	-0.500793	1.270081	0.226080	1.326978	1.143574	0.226080	1.326978	1.143574
H	-0.247865	1.290365	1.165848	1.112198	2.143321	-0.249298	1.112198	2.143321	-0.249298

Table S3. Cartesian coordinates of the reactant, TS and product for reaction C.

	Reactant			TS			Product		
	X	Y	Z	X	Y	Z	X	Y	Z
C	2.299391	-0.109170	0.000000	0.881619	1.128480	-0.115927	-0.997574	-0.358970	-0.076658
H	2.565960	0.941372	0.000000	0.243570	1.371369	-0.955577	0.031610	0.762651	-0.228645
C	1.031230	-0.507370	0.000000	1.326855	-0.187747	0.062384	-0.333090	1.689526	0.214359
O	0.000000	0.402548	0.000000	0.560962	-1.163470	-0.203066	-2.166218	-0.159502	0.140063
N	-1.288742	-0.286361	0.000000	-0.957830	-0.012769	0.493746	1.336393	0.372011	0.407438
O	-2.166493	0.491592	0.000000	-1.870293	0.101889	-0.185103	1.807022	-0.619365	-0.107528
H	0.709683	-1.545567	0.000000	1.402235	1.957794	0.354621	-0.570130	-1.376449	-0.192627
H	3.093774	-0.845157	0.000000	2.282801	-0.391531	0.571345	0.217821	0.891698	-1.302266

Table S4. Cartesian coordinates of the reactant, TS and product for reaction D.

	Reactant			TS			Product		
	X	Y	Z	X	Y	Z	X	Y	Z
C	-1.251673	0.435295	0.057426	1.298889	-0.130448	-0.040051	1.208876	0.315598	0.000000
O	0.059090	0.764803	-0.092311	0.532072	-1.147106	-0.048447	1.439776	-0.866610	0.000000
N	0.988209	-0.404801	0.256220	-0.961023	0.049551	0.483429	-1.336852	0.346568	0.000001
O	2.058246	-0.161389	-0.118687	-1.799313	0.082843	-0.280005	-1.363290	-0.854496	0.000000
H	-1.827071	1.331561	0.317124	2.386816	-0.297494	0.025692	1.996411	1.097102	0.000002
N	-1.689271	-0.738864	-0.118115	0.761859	1.057161	-0.099514	-0.056157	0.917622	-0.000002
H	-2.694151	-0.765000	0.059564	1.351917	1.847301	0.154828	-0.110486	1.928836	0.000001

Table S5. Cartesian coordinates of the reactant, TS and product for reaction E.

	Reactant			TS			Product		
	X	Y	Z	X	Y	Z	X	Y	Z
C	1.208642	0.377470	0.084853	1.361672	-0.109445	-0.087399	1.111830	0.511601	-0.000001
N	-1.251638	0.436335	0.225763	-0.968298	-0.016838	-0.485315	-1.378121	0.243658	0.000000
O	-2.121178	-0.402120	0.239564	-1.914357	0.059735	0.164772	-1.243193	-0.971013	-0.000001
N	2.146469	-0.478011	0.145964	2.250075	-0.233913	-0.717448	1.797479	1.354655	-0.000006
H	1.247390	1.383278	0.525741	0.839750	1.172143	0.137840	-0.220690	0.949913	0.000001
H	2.949160	-0.092436	0.652592	0.237947	1.364336	1.016584	-0.402235	1.943704	0.000006
C	-0.074270	0.044779	-0.628529	1.267084	2.037582	-0.358784	1.579251	-0.743219	0.000001
H	-0.169630	0.691542	-1.514716	0.603073	-1.125100	0.274822	0.940796	-1.611519	0.000004
H	-0.127546	-1.007182	-0.920167	0.907791	-2.028503	-0.087721	2.654694	-0.884021	-0.000001

Table S6. Cartesian coordinates of the reactant, TS and product for reaction F.

	Reactant			TS			Product		
	X	Y	Z	X	Y	Z	X	Y	Z
C	-1.822165	-0.624870	-0.027315	-0.265252	-0.847851	1.132176	-0.076851	0.771523	0.074019
H	-2.900049	-0.737062	-0.157115	0.287198	-0.915852	2.066056	-0.462572	1.397106	-0.740096
C	0.076851	0.771523	0.074019	-0.265252	-0.847851	-1.132176	1.822165	-0.624870	-0.027315
H	0.240226	1.321106	1.012964	-1.356915	-0.821939	-1.199536	1.242502	-1.535651	0.147457
H	-1.242502	-1.535651	0.147457	-1.356915	-0.821939	1.199536	-0.240226	1.321106	1.012964
N	0.942134	-0.458103	0.186985	-0.265252	1.000530	0.000000	-0.942134	-0.458103	0.186985
O	2.083451	-0.243558	-0.154044	0.560293	1.809879	0.000000	-2.083451	-0.243558	-0.154044
H	0.462572	1.397106	-0.740096	0.287198	-0.915852	-2.066056	2.900049	-0.737062	-0.157115
N	-1.335844	0.547109	-0.088569	0.385267	-1.118993	0.000000	1.335844	0.547109	-0.088569

Table S7. Cartesian coordinates of the reactant, TS and product for reaction G.

	Reactant			TS			Product		
	X	Y	Z	X	Y	Z	X	Y	Z
C	1.496388	-0.730681	0.000000	0.365324	0.787726	-0.386023	1.964338	0.399649	0.590678
H	1.301684	-1.801062	-0.000001	-0.371391	1.096355	-1.119016	2.901190	0.192976	0.072003
C	0.418016	0.064709	-0.000003	0.540911	-0.601000	-0.235335	1.734044	1.468528	0.525854
C	0.373326	1.562405	-0.000003	1.815362	-1.235191	0.271158	2.066570	0.149507	1.653378
H	-0.157881	1.943573	-0.878433	1.841711	-2.280092	-0.045970	0.844532	-0.399835	-0.035503
H	-0.157875	1.943574	0.878431	2.712052	-0.720169	-0.082561	-0.561032	-0.213516	0.556015
H	1.383219	1.971598	-0.000006	1.827058	-1.225738	1.368902	-0.508810	-0.089554	1.647327
O	-0.799432	-0.618933	-0.000009	-0.488801	-1.349272	-0.372760	-1.533057	-1.340253	0.201869
N	-1.967055	0.225581	0.000009	1.342760	1.833001	0.065352	-1.230329	-2.267879	0.693378
O	-2.941700	-0.440654	0.000001	1.965794	1.489988	0.895932	-2.544652	-1.083182	0.525659
C	2.936418	-0.313528	0.000004	2.015096	2.141876	-0.749168	-1.527253	-1.511828	-0.876238
H	3.071326	0.770107	0.000001	0.819168	2.737483	0.394423	1.020672	-1.152901	-0.972387
H	3.456535	-0.713793	0.879251	-1.564005	0.026808	0.544890	-1.235627	1.079980	0.111871
H	3.456542	-0.713799	-0.879237	-2.542148	0.332450	0.026799	-0.586929	1.766063	-0.640464

Table S8. Cartesian coordinates of the reactant, TS and product for reaction H.

	Reactant			TS			Product		
	X	Y	Z	X	Y	Z	X	Y	Z
C	-0.460498	0.023223	-0.000001	0.525265	-0.519231	-0.070907	1.030184	-0.383584	0.000000
O	0.774207	-0.638752	-0.000003	-0.533754	-1.272636	-0.016751	1.232715	-1.575595	0.000000
N	1.928706	0.235513	0.000003	-1.648028	0.110651	0.477160	-1.306194	-0.787648	0.000000
O	2.912797	-0.409871	0.000000	-2.496942	0.251421	-0.272309	-2.424249	-0.321437	0.000001
N	-1.434839	-0.777888	0.000000	0.316674	0.759540	-0.155082	-0.288500	0.156687	0.000000
C	-0.417188	1.529254	-0.000001	1.857211	-1.233582	-0.001925	2.142980	0.645678	0.000001
H	-1.425035	1.941760	-0.000002	2.646546	-0.684919	-0.519554	2.092623	1.290460	-0.883219
H	0.117172	1.901347	0.878451	2.154528	-1.365749	1.044101	2.092619	1.290463	0.883217

H	0.117174	1.901346	-0.878452	1.753388	-2.224031	-0.448707	3.089626	0.106732	0.000003
C	-2.814115	-0.345594	0.000001	1.324088	1.776884	0.028461	-0.592747	1.585917	0.000000
H	-2.985672	0.739419	0.000000	2.351851	1.387836	0.008307	-0.191559	2.072269	-0.892663
H	-3.312969	-0.769782	-0.878501	1.233433	2.538828	-0.753454	-1.679115	1.672730	-0.000004
H	-3.312967	-0.769780	0.878507	1.185920	2.281993	0.993468	-0.191566	2.072267	0.892666

Table S9. Cartesian coordinates of the reactant, TS and product for reaction I.

	Reactant			TS			Product		
	X	Y	Z	X	Y	Z	X	Y	Z
C	1.996788	-0.892727	0.036188	0.470124	1.377600	-0.125719	-1.033524	-0.113525	-0.005786
H	1.837052	-1.961039	0.003489	-0.351479	1.703481	-0.745682	0.351178	-0.651294	-0.406117
C	0.951819	-0.053049	-0.003991	0.850290	0.045837	-0.153528	0.332026	-0.817508	-1.491900
O	-0.306048	-0.627307	-0.038189	-0.027698	-0.876043	-0.448797	-1.941931	-0.879417	0.263020
N	-1.401570	0.324908	0.047211	-1.349647	-0.129778	0.540926	1.416216	0.359472	-0.175616
O	-2.430475	-0.244454	-0.008112	-2.336140	-0.058040	-0.040922	2.395993	-0.079870	0.383142
H	3.010425	-0.513485	0.070644	1.104876	2.137398	0.316850	0.564549	-1.590242	0.110078
N	0.998672	1.330519	-0.100073	2.080466	-0.399824	0.277726	-1.142929	1.240265	-0.025397
H	0.258436	1.826493	0.376880	2.236647	-1.391914	0.174892	-2.018926	1.652068	0.258682
H	1.914916	1.708789	0.096246	2.882443	0.190295	0.116609	-0.329070	1.820733	-0.167657

Table S10. Cartesian coordinates of the reactant, TS and product for reaction J.

	Reactant			TS			Product		
	X	Y	Z	X	Y	Z	X	Y	Z
C	-1.618174	-1.531482	0.000159	0.141220	1.294081	0.270147	-0.638396	-0.583579	0.110037
H	-1.280769	-2.560889	0.000150	0.955941	1.486774	0.955642	0.673555	-0.202920	0.757244
C	-0.732428	-0.527532	-0.000040	-0.501693	0.048115	0.318898	0.997525	-1.036354	1.389202
O	0.612755	-0.804464	-0.000333	0.151876	-1.014909	0.550335	-0.875629	-1.690317	-0.321525
N	1.470720	0.432194	0.000301	1.631724	-0.153617	-0.596687	1.765454	-0.131317	-0.341092
O	2.591155	0.122561	0.000002	2.680403	-0.126540	-0.157069	2.424887	0.863891	-0.255884
H	-2.679914	-1.322943	0.000303	-0.336970	2.153285	-0.188554	0.646506	0.740385	1.306535
C	-1.158703	0.848350	-0.000045	-1.900083	-0.064626	-0.090350	-1.625235	0.505219	-0.019473
N	-1.558543	1.939668	-0.000052	-3.019419	-0.156939	-0.389797	-2.407975	1.359182	-0.110810

Table S11. Cartesian coordinates of the reactant, TS and product for reaction K.

	Reactant			TS			Product		
	X	Y	Z	X	Y	Z	X	Y	Z
C	1.099681	-0.804109	-0.200030	0.826373	-0.810637	0.303230	0.287040	0.387879	0.334088
H	0.569912	-1.704276	-0.494592	0.189887	-1.521144	0.812787	0.559412	0.297489	1.391120
C	0.449641	0.374651	-0.185712	0.437323	0.497636	0.206990	-0.973061	-0.448129	0.063291
O	-0.904541	0.440427	-0.495346	-0.823016	0.855409	0.426688	-0.897682	-1.662543	-0.040612
N	-1.721046	-0.378475	0.433181	-1.661422	-0.240750	-0.523060	1.407710	-0.212801	-0.502869
O	-2.835447	-0.423053	0.064812	-2.655631	-0.558779	-0.015514	2.339320	-0.604236	0.158680
N	1.099741	1.582132	0.172138	1.303744	1.486079	-0.280486	-2.115218	0.277457	-0.024260
H	0.964406	2.294252	-0.542977	0.878099	2.404386	-0.244449	-2.990482	-0.205407	-0.155347
H	0.721717	1.960729	1.039772	2.215792	1.477686	0.165555	-2.084445	1.277881	0.105929
N	2.410986	-0.971358	0.214874	2.114012	-1.221757	-0.062734	0.141403	1.781154	-0.036013
H	2.944594	-1.651718	-0.309850	2.231326	-2.225480	-0.094313	0.915813	2.333476	0.322636
H	2.895569	-0.087322	0.325020	2.437568	-0.795483	-0.926324	0.145454	1.871620	-1.051166

Table S12. Cartesian coordinates of the reactant, TS and product for reaction L.

	Reactant			TS			Product		
	X	Y	Z	X	Y	Z	X	Y	Z
C	1.191724	-1.046859	0.000001	0.058186	0.679555	0.526791	0.436889	0.517873	0.562647
H	1.065737	-2.124906	0.000001	0.810018	0.942356	1.262008	1.013573	0.588311	1.488626
C	0.085119	-0.264803	0.000004	-0.183867	-0.709089	0.320753	-0.212561	-0.823593	0.457096
O	-1.131068	-0.859086	0.000010	0.771669	-1.524323	0.369166	0.366037	-1.822516	0.837459
N	-2.320441	0.160869	-0.000011	1.905924	-0.033219	-0.608892	1.751514	0.538150	-0.573182
O	-3.310261	-0.426067	-0.000003	2.913643	0.235721	-0.169448	2.254828	-0.505344	-0.705198
C	0.163018	1.172384	0.000003	-1.503200	-1.183759	-0.080790	-1.504455	-0.912343	-0.240061
C	2.512910	-0.530795	-0.000003	-2.547707	-1.596224	-0.379148	-2.526885	-1.025380	-0.779892
N	3.611833	-0.145775	-0.000005	-0.846006	1.697618	0.139323	-0.351932	1.696295	0.299552
N	0.244076	2.331558	0.000003	-1.564387	2.552372	-0.197132	-0.965792	2.653685	0.064200

Table S13. Cartesian coordinates of the reactant, TS and product for reaction M.

	Reactant			TS			Product		
	X	Y	Z	X	Y	Z	X	Y	Z
C	-1.197505	-0.863536	-0.015292	0.533327	-0.518139	0.421320	0.511327	-0.978300	0.010890
H	-1.040420	-1.933095	-0.044793	-0.021689	-1.182136	1.068740	-0.287068	0.313691	0.390927
C	-0.119854	-0.024852	-0.013924	0.030637	0.778267	0.197638	-0.073906	0.559104	1.437747
O	1.103916	-0.623791	0.000711	-1.248084	0.985889	0.294154	-0.038515	-1.936519	-0.488903
N	2.257383	0.335957	0.020017	-1.765681	-0.544973	-0.566646	0.196875	1.530289	-0.438687
O	3.257622	-0.263230	0.008698	-2.596130	-1.084835	-0.000928	1.323754	1.847195	-0.158975
C	-2.506976	-0.336009	0.007719	1.859237	-0.857480	0.080826	-1.719636	0.151898	0.187584
N	-3.567823	0.151959	0.032462	2.961363	-1.098657	-0.225655	-2.867201	0.075006	0.043212
N	-0.177810	1.334282	-0.078215	0.811120	1.811364	-0.191281	1.826692	-0.905200	0.342051
H	-1.094389	1.744365	0.032444	0.373448	2.660084	-0.510992	2.420769	-1.652001	0.010886
H	0.606274	1.875890	0.246206	1.815128	1.723599	-0.217196	2.248929	-0.016915	0.571960

Table S14. Cartesian coordinates of the reactant, TS and product for reaction N.

	Reactant			TS			Product		
	X	Y	Z	X	Y	Z	X	Y	Z
C	-0.925631	-1.186444	-0.136380	0.147978	0.883925	0.451432	0.628119	-0.671125	-0.062297
H	-0.340858	-2.080564	-0.328505	0.973952	1.039757	1.133752	-0.697965	-0.188358	0.469300
C	-0.308084	0.018943	-0.270367	-0.412905	-0.429274	0.393971	-0.583917	0.381745	1.396440
O	1.018133	0.091785	-0.660966	0.278905	-1.458700	0.583870	0.752890	-1.706783	-0.685103
N	1.934672	-0.147903	0.515232	1.719705	-0.321245	-0.657375	-1.237280	0.866143	-0.613585
O	3.045194	-0.112934	0.162493	2.786385	-0.156303	-0.256326	-1.258734	1.987795	-0.198125
N	-2.233282	-1.384176	0.170587	-0.539751	2.006863	0.073971	-1.633299	-1.249288	0.559449
H	-2.546577	-2.300888	0.441178	-1.263221	1.914587	-0.626101	-2.587395	-0.928368	0.680338
H	-2.796919	-0.601800	0.471206	-0.041123	2.882666	0.055039	-1.561745	-1.880745	-0.232328
C	-0.998809	1.240566	-0.033117	-1.784677	-0.543402	-0.110316	1.767574	0.233944	0.148773
N	-1.619550	2.205515	0.177478	-2.879140	-0.597687	-0.501392	2.669642	0.944931	0.323667
